# Putative adverse outcome pathways for female reproductive disorders to improve testing and regulation of chemicals

**DOI:** 10.1007/s00204-020-02834-y

**Published:** 2020-07-07

**Authors:** Hanna K. L. Johansson, Pauliina Damdimopoulou, Majorie B. M. van Duursen, Julie Boberg, Delphine Franssen, Marijke de Cock, Kersti Jääger, Magdalena Wagner, Agne Velthut-Meikas, Yuling Xie, Lisa Connolly, Pauline Lelandais, Severine Mazaud-Guittot, Andres Salumets, Monica Kam Draskau, Panagiotis Filis, Paul A. Fowler, Sofie Christiansen, Anne-Simone Parent, Terje Svingen

**Affiliations:** 1 Division of Diet, Disease Prevention and Toxicology, National Food Institute, Technical University of Denmark, 2800 Kongens Lyngby, Denmark; 2 Department of Clinical Science, Intervention and Technology, Karolinska Institutet and Karolinska University Hospital, 14186 Stockholm, Sweden; 3 Department Environment and Health, Vrije Universiteit Amsterdam, De Boelelaan 1085, 1081 HV Amsterdam, The Netherlands; 4 Neuroendocrinology Unit, GIGA Neurosciences, University of Liège, Sart-Tilman, 4000 Liège, Belgium; 5 Faculty of Science, Vrije Universiteit Amsterdam, 1081 HV Amsterdam, The Netherlands; 6 Competence Centre on Health Technologies, Tartu, Estonia; 7 Department of Chemistry and Biotechnology, School of Science, Tallinn University of Technology, Tallinn, Estonia; 8 Institute for Global Food Security, School of Biological Sciences, Queen’s University Belfast, Belfast, BT9 5DL Northern Ireland UK; 9 Inserm, EHESP, Irset (Institut de Recherche en Santé, Environnement et Travail) UMR_S 1085, University Rennes, 35000 Rennes, France; 10 Department of Obstetrics and Gynaecology, Institute of Clinical Medicine, University of Tartu, Tartu, Estonia; 11 Department of Obstetrics and Gynecology, University of Helsinki, Helsinki University Hospital, Helsinki, Finland; 12 Institute of Medical Sciences, University of Aberdeen, Foresterhill, Aberdeen, AB25 2ZD UK; 13 Department of Pediatrics, CHU de Liège, Rue de Gaillarmont 600, 4032 Liège, Belgium

**Keywords:** Ovary, Reproduction, Adverse outcome pathway, AOP, Ovarian dysgenesis syndrome, ODS, Endocrine-disrupting chemicals, EDC

## Abstract

Modern living challenges female reproductive health. We are witnessing a rise in reproductive disorders and drop in birth rates across the world. The reasons for these manifestations are multifaceted and most likely include continuous exposure to an ever-increasing number of chemicals. The cause–effect relationships between chemical exposure and female reproductive disorders, however, have proven problematic to determine. This has made it difficult to assess the risks chemical exposures pose to a woman’s reproductive development and function. To address this challenge, this review uses the adverse outcome pathway (AOP) concept to summarize current knowledge about how chemical exposure can affect female reproductive health. We have a special focus on effects on the ovaries, since they are essential for lifelong reproductive health in women, being the source of both oocytes and several reproductive hormones, including sex steroids. The AOP framework is widely accepted as a new tool for toxicological safety assessment that enables better use of mechanistic knowledge for regulatory purposes. AOPs equip assessors and regulators with a pragmatic network of linear cause–effect relationships, enabling the use of a wider range of test method data in chemical risk assessment and regulation. Based on current knowledge, we propose ten putative AOPs relevant for female reproductive disorders that can be further elaborated and potentially be included in the AOPwiki. This effort is an important step towards better safeguarding the reproductive health of all girls and women.

## Introduction

A woman’s reproductive health affects numerous aspects of her life, from the ability to conceive to personal well-being and social standing. It is also of paramount importance for society more broadly, as it has direct consequences for overall birth rates, health of offspring, and national health budgets. Therefore, any factor that can negatively influence female reproductive development or function should be of utmost concern and efforts put in place to minimize potentially harmful effects. In recent years, a growing body of evidence points towards an association between exposure to chemicals and female reproductive disorders, with certain life stages suggested to be particularly vulnerable to xenobiotic insult.

A current consensus is that many female reproductive disorders such as infertility (see Table 1 for definitions), polycystic ovary syndrome (PCOS), premature (or primary) ovarian insufficiency (POI), and early menopause have a common origin; disrupted ovarian development or function during development, collectively referred to as the ovarian dysgenesis syndrome (ODS) hypothesis (Buck Louis et al. 2011; Johansson et al. 2017). This is analogous to other ‘developmental origins of health and diseases (DOHaD)’ hypotheses, whereby disrupted tissue development or function can have long-lasting consequences for health and disease. It is easy to see how disrupted ovarian development can have lifelong consequences, since a fully functional reproductive system hinges on events occurring already during fetal life. It is more difficult, however, to determine causal relationships between disturbed development in early life and reproductive disorders later in life; especially in humans. This is due, not only, to the significant timespan between early initiating events and time of disease manifestations, but also to the sheer complexity of reproductive development, from the intrinsic regulatory aspects of genetic and hormonal control to the extrinsic influences posed by the environment.

**Table 1:** Definition of terms related to female reproductive medicine

Term	Definition
Fertility	Refers to actual output of reproduction. That is, the actual number of children born to a woman
Fecundity	Refers to the capacity to conceive given unprotected intercourse
Subfertility	Reduced fertility, i.e., still possible to conceive, but takes longer than average despite regular unprotected sexual intercourse
Infertility	The failure to achieve a clinical pregnancy after 12 months or more of regular unprotected sexual intercourse
Sterility	A physiological inability to achieve pregnancy

The terms used to denote female reproductive capacity and disorders are somewhat ambiguous and a general consensus does not exist (Habbema et al. 2004; Jenkins et al. 2004; Vander Borght and Wyns 2018). To avoid confusion, for the purposes of this review the following terms are defined as follows

Environmental factors are undoubtedly contributing to a rise in female reproductive disorders, likely also including developmental exposure to industrial chemicals (Fowler et al. 2012; Hunt et al. 2016; Johansson et al. 2017). A classic example of an endocrine-disrupting chemical (EDC) causing female reproductive disease is the pharmaceutical diethylstilbestrol. Prescribed to women to alleviate pregnancy complications in the 1940–1970s, intrauterine exposure to diethylstilbestrol turned out to have the disastrous consequence of causing vaginal cancers in daughters once they reached young adulthood (Reed and Fenton 2013). Following the discovery that fetal exposure to diethylstilbestrol could also cause additional female reproductive disorders in adulthood, there has been a multitude of reproductive disorders associated with fetal exposure to chemicals more broadly. This includes compromised fecundity (Table 1) after exposure to diethylstilbestrol (Palmer et al. 2001) or cigarette smoke (Jensen et al. 2006), PCOS after exposure to bisphenol A (Palioura and Diamanti-Kandarakis 2015), POI after exposure to perfluoroalkyl substances (Zhang et al. 2018), premature menopause after exposure to diethylstilbestrol (Hatch et al. 2006), and more as discussed later in this review.

According to a recent estimate, as many as 330,000 chemicals are, or have been, in commerce (Wang et al. 2020). Because of the growing body of evidence suggesting that chemical exposures can disrupt female reproductive health, there is a clear need to better protect women, not least the unborn child, against harmful chemicals. With this comes a growing requirement to construct a better chemical testing strategy that can detect compounds of concern at an early stage of substance evaluation, ideally before they are introduced onto the market. One such strategy can be based on the adverse outcome pathway (AOP) framework, used as a pragmatic tool to inform chemical safety assessment strategies, as described in organization for economic co-operation and development (OECD) guidance document 184 (OECD 2017).

In this review, we will use the AOP concept to highlight current knowledge and key knowledge gaps pertaining to several female reproductive disorders. This will guide our efforts to elaborate new and improved test strategies for the detection of chemicals disrupting female reproductive development and function. First, we will provide an overview of female reproductive pathologies related to ovary function to define the scope of our work. In the second half of this review, we propose ten putative AOPs (pAOP) relevant for female reproductive disease outcomes, including scientific rationale for the various pAOPs. This work is an important first step in the EU Horizon 2020 project FREIA: *A human evidence-based screening and identification approach for female reproductive toxicity of endocrine-disrupting chemicals* [(van Duursen et al. 2020); https://www.freiaproject.eu]. To maintain a close focus in the current review, we will address pAOPs related to disrupted pubertal onset, another component in the establishment of fertility in women, in a separate publication.

## Ovarian pathologies

Most women who suffer from ovarian disorders will also be burdened with fertility issues. This does not mean that all subfertile women also suffer from ovarian dysgenesis, but we may speculate that many do, a view that is increasingly substantiated by research (Axmon et al. 2000; Buck Louis et al. 2012; Ehrlich et al. 2012; Pizzorno 2018). If many ovarian dysgenesis cases arise from developmental exposure to industrial chemicals, the burden to our health costs becomes sizeable. Although they vary between studies, prevalence figures for infertility are high. A Dutch study found a prevalence of 2.2% between 1985 and 2006 when defining infertility as an inability to conceive for 2 years (van de Lisdonk et al. 2008). A study modelling worldwide infertility trends between 1990–2010 using data from 277 surveys from 190 countries, on the other hand, reported a prevalence of 1.9% for primary infertility, defined as *the inability to conceive a first child defined as ‘no live births for at least 5 years without the use of contraception* (Mascarenhas et al. 2012).

Several ovarian disorders may first manifest after puberty, particularly if they have clinical signs related to the menstrual cycle. The most common disorders diagnosed in young women of reproductive age are PCOS, ovarian cysts and endometriosis. PCOS is a collection of disorders and is characterized by biological or clinical hyperandrogenism, oligo- or anovulation, and the presence of an excess number of ovarian cysts. These cysts are actually immature follicles, each trapping an egg (Ibáñez et al. 2017). This accumulation of follicles will typically reduce the number of eggs available to be released from the ovaries (oligo-ovulation and anovulation) and affected women may experience irregular menses. The prevalence of PCOS varies depending on the diagnostic criteria used and the populations sampled. A 2016 meta-analysis of studies grouped based on diagnostic guidelines (by NIH, Rotterdam, or The Androgen Excess-PCOS Society) reported a prevalence of 6–10% of women (Bozdag et al. 2016). Androgen excess is a key characteristic of PCOS and observed in 60–80% of patients, but there are also other diagnostic criteria such as anovulation or ultrasound confirmation of polycystic ovarian morphology (Goldrat and Delbaere 2018; Ibáñez et al. 2017). Though the etiology of PCOS remains unclear, it has been suggested that overexposure to androgens in utero may increase the risk for developing PCOS later in life (Filippou and Homburg 2017). Although human studies on prenatal exposure to EDCs and PCOS are lacking, there are some studies that have compared levels of EDCs in PCOS patients. A meta-analysis of 11 case–control studies reported that women with PCOS had higher levels of bisphenol A compared to controls without PCOS (Hu et al. 2018). Similar results were found for levels of perfluorooctanoic acid and perfluoro-octanesulfonate in PCOS patients (Vagi et al. 2014).

Endometriosis is a non-malignant condition, whereby tissue normally lining the uterine cavity also grows on the outer surface of the uterus, and even on the ovaries and elsewhere in the peritoneal cavity (Vercellini et al. 2014). It may cause the formation of cysts known as endometriomas, a painful condition associated with an increased risk for infertility (Eisenberg et al. 2018; Prescott et al. 2016). Prevalence rates of endometriosis vary greatly between studies, but, in the general population, it is estimated to affect around 0.7–8.6% of women. Strikingly, the prevalence of endometriosis is up to 70% among infertile women (Ghiasi et al. 2020). There is no consensus on the etiology of endometriosis, but it is considered an estrogen-dependent disease (Kitawaki et al. 2002), thus endocrine disruptors are regarded as potential causal factors (Crain et al. 2008). This is also why we include endometriosis under ‘ovarian dysgenesis disorders’, as it may arise from early disruption to ovary development. For example, prenatal exposure to diethylstilbestrol leads to an 80% increased risk of developing endometriosis in women from the National Health and Nutrition Examination Survey (NHANES II) cohort who had not previously been diagnosed with infertility or cancer (Missmer et al. 2004). In addition, associations between endometriosis and various chemicals have been observed in case–control studies, including polychlorinated biphenyls, polybrominated diphenyl ethers, and various organochlorine pesticides (Ploteau et al. 2017; Upson et al. 2013).

Other indications of ovarian dysfunction may first become apparent in adulthood, when women wish to conceive. These include infertility and POI. A woman is considered to have POI if she is under 40 years of age, has had amenorrhea (absence of menstrual bleeding) for more than 4 months and two elevated serum follicle-stimulating hormone (FSH) measurements within the menopausal range occurring at least 1 month apart (Nelson 2009). There are multiple established etiologies, but over 90% of POI cases are idiopathic without any obvious cause (Nelson 2009; Torrealday et al. 2017). Women with POI may experience symptoms similar to menopause as a result of low estrogen levels. Unlike postmenopausal women, however, they may still become pregnant, albeit with a reduced chance of conceiving (Fraison et al. 2019). The prevalence of POI is generally estimated to be 1%, but a study using Swedish Patient Registry data from 1973 to 1993, including just over 1 million women, reported a prevalence of 1.9% (Lagergren et al. 2018). Furthermore, based on a recent meta-analysis of 31 studies, a pooled prevalence of 3.7% was found (Golezar et al. 2019). A few studies report on associations between chemical exposure levels and POI. Exposure to perfluorinated alkyl substances was associated with a higher risk for POI in Chinese women (Zhang et al. 2018), whereas a cross-sectional study on women from the USA looking at over 100 EDCs found associations between early menopause and numerous compounds, including phthalates, polychlorinated biphenyls and organochlorine pesticides (Grindler et al. 2015). Finally, tobacco smoke has been shown by several studies to correlate negatively with both POI and early menopause, as reviewed by others (Vabre et al. 2017).

It is clear from the above-mentioned literature that female reproductive health is challenged by developmental exposure to chemicals. This view is further substantiated by a large number of animal studies, as will be discussed later. The challenge is that, despite clear associations between exposure and disease manifestations, we lack essential fundamental knowledge at the more mechanistic level about how these diseases develop and how chemical stressors can initiate disease development. This is exactly the type of information we need if we are to more rapidly, and with better precision, screen a large number of potentially harmful chemicals and thereby regulate their use before they cause health problems. One vision is to amass enough mechanistic knowledge that we can elaborate into robust safety testing strategies making use of both animal and non-animal data when testing chemicals. One strategy to achieve this is by the structured gathering of our knowledge using the AOP framework and identifying needs for test method development. This is the incentive for the work presented in this review. In the following, we will briefly introduce the AOP concept before continuing with elaborating a number of putative AOPs (pAOP). We believe that these pAOPs are a good starting point for further development aimed at assisting chemical risk assessment and regulation.

## The adverse outcome pathway (AOP) concept in brief

An AOP describes a sequence of events from an initial molecular ‘trigger’ through to an adverse outcome (AO) in an intact organism. An AOP does not aim to cover all events that take place in a complex biological system challenged by a chemical, nor all the steps towards an adverse effect, but rather focusses on a minimum number of key events (KEs) that can help explain cause–effect relationships and at the same time be measurable. Central to the AOP thinking is that the AOs should be of relevance for chemical safety assessment or regulatory purposes (Ankley and Edwards 2018). To enable the scientific community to share, develop and discuss their AOP-related knowledge, the OECD has launched the "Adverse Outcome Pathway Knowledge Base (AOP-KB)", which includes the AOPwiki platform, where AOPs can be entered and information about links between MIEs, KEs, and AOs presented (https://aopkb.oecd.org/background.html).

As described in the OECD User Handbook (OECD 2018b), a typical AOP (see Table 2 for key component definitions) starts with a molecular initiating event (MIE), which describes the first chemical interaction with a biomolecule. This MIE, or ‘trigger’, then leads to a downstream KE: defined as *a change in biological or physiological state that is both measurable and essential to the progression towards a specific AO*. The AO is in itself a KE, but of regulatory significance and typically describes an apical endpoint: an observable effect in an intact organism, such as a clinical or pathological sign, that is indicative of a disease state resulting from exposure to a toxicant (Krewski et al. 2010). Finally, the individual KEs in an AOP should be linked by key event relationships (KERs), which refer to biologically plausible and predictive relationships between two KEs.

**Table 2:** Definition of terms used to describe an adverse outcome pathway (AOP); adapted from (OECD 2018b)

Term	Abbreviation	Definition
Molecular initiating event	MIE	The initial point of interaction between a chemical/stressor and a biomolecule within an organism that will result in a disruption capable of initiating the AOP
Key event	KE	A change in biological or physiological state that is both measurable and necessary to progress through the AOP
Key event relationship	KER	A biologically plausible and scientifically based relationship between two KEs, describing a causal and predictive connection between events that can facilitate extrapolation of the state of a downstream event from known (measured or predicted) upstream events
Adverse outcome	AO	A KE describing a change to a biological or physiological state that is generally regarded of regulatory significance or equivalent to an apical endpoint in an accepted regulatory guideline toxicity test
Apical endpoint		An observable effect in an intact organism, such as a clinical or pathological sign, that is indicative of a disease state resulting from exposure to a toxicant. In essence, this is an AO

**Fig. 1:**
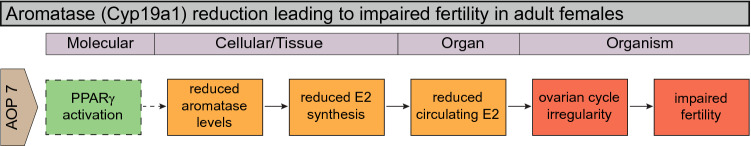
AOP7 from the AOPwiki describing PPARγ-mediated reduction in aromatase as a MIE leading to irregular ovarian cycling and impaired fertility in adult females. Activation of the peroxisome proliferator activated receptor gamma (PPARγ) can impair steroidogenesis via reduced aromatase level (expression or activity), which causes reduced circulating estradiol (E2) levels. This will ultimately affect ovarian cycling and fertility. The AOP is mainly based on rodent data, with supporting evidence from human epidemiological studies. Adapted from the AOPwiki at https://aopwiki.org/aops/7

**Fig. 2:**
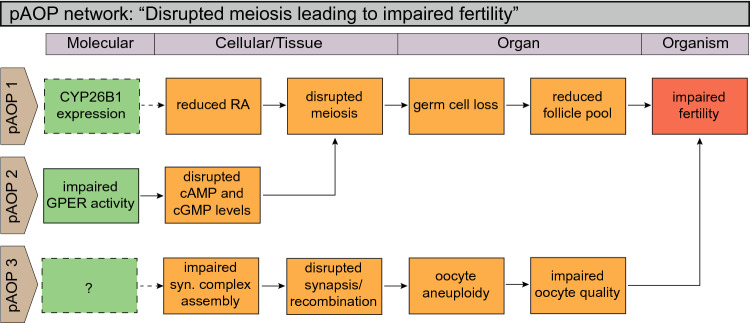
Putative AOP (pAOP) network for perturbed meiosis leading to impaired fertility in females. pAOP 1: germ cell meiosis in females initiates during fetal life in humans. Retinoic acid is a critical factor initiating meiosis in the fetal ovaries. Ectopic activation, or maintained expression, is hypothesized to prevent retinoic acid-dependent activation in the ovaries. pAOP 2: initiation and maintenance of meiotic arrest requires high levels of cAMP mediated through G-protein-coupled receptors (GPCRs) such as GPER. By blocking, e.g., GPER, meiosis can be disrupted by affecting cAMP/cGMP levels. pAOP 3: the proper assembly of the synaptonemal (syn.) complex is necessary for meiosis, a process that can be disrupted by chemical exposures. The molecular triggers are yet to be characterized

**Fig. 3:**
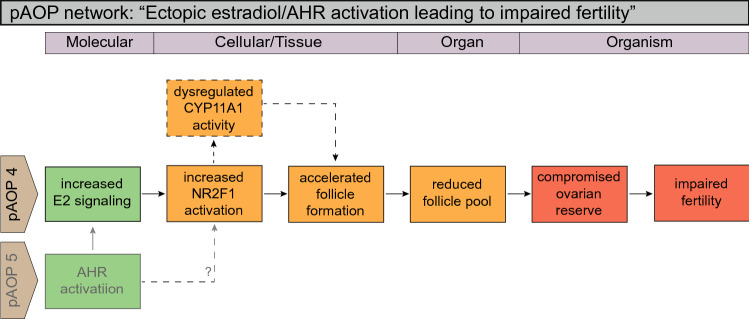
Putative AOP (pAOP) network for disrupted follicle formation leading to impaired fertility in females. Follicle assembly is essential for adult fertility and depends on a coordinated sequence of cell proliferation and differentiation, collection of primordial granulosa cells around germ cells in meiotic arrest, as well as germ cell death. pAOP 4: aryl hydrocarbon receptor (AHR) and estrogen receptor α (ERα) signaling are known to be involved in regulating these processes. AHR activation can also activate other downstream effects, for example as proposed in pAOP 5 (Fig. 4). The AOP is based primarily on human fetal ovary studies, supported by animal, experimental and human epidemiological studies

**Fig. 4:**
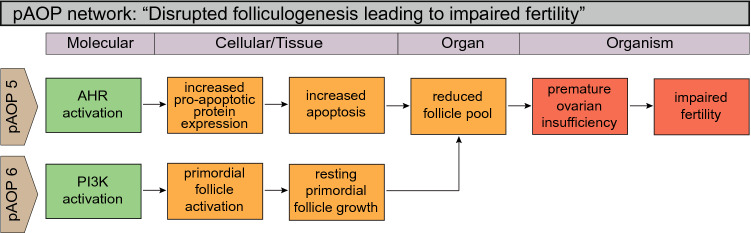
Putative AOP (pAOP) network for disrupted folliculogenesis leading to impaired fertility in females. Folliculogenesis is a complex process regulated by several signaling pathways. As suggested, the disruption to key signaling molecules such as pAOP 5 AHR or pAOP 6 PI3K can affect the ovarian follicles by separate cellular events, ultimately reducing the availability of competent follicles. In turn, this can affect female physiology and fertility

**Fig. 5:**
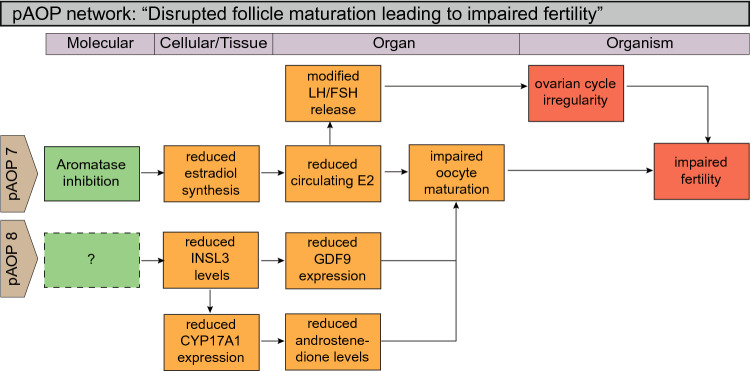
Putative AOP (pAOP) network for impaired steroidogenesis/follicle maturation leading to impaired fertility in females. Steroidogenesis is intimately linked to reproductive function and disrupted steroid synthesis or action can impair fertility by many different mechanisms. pAOP 7: aromatase (CYP19A1) action is highlighted as being central to female reproduction, as enzymatic activity is necessary for estrogen (estradiol) synthesis, which in turn is central for the regulation of oocyte maturation, but also ovarian cyclicity. pAOP 8: insulin-like factor 3 (INSL3), another hormone synthesized by theca cells, is also important for oocyte maturation, with knockout mice displaying an infertility phenotype or increased follicle apoptosis. The upstream MIE remains elusive and could also include compromised theca cell differentiation or function

**Fig. 6:**
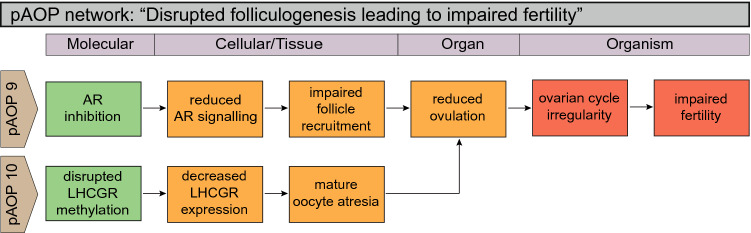
Putative AOP (pAOP) network for disrupted folliculogenesis leading to impaired fertility in adult females. Similarly to pAOPs 5, 6 (Fig. 4), folliculogenesis can be disrupted through many different key signaling pathways. In adult females, this includes pAOP 9 AR and pAOP 10 LHCGR, which, upon disruption, could negatively impact follicle recruitment or maturation, ultimately causing irregular ovarian cycling and subfertility

Once an AO has been defined, and specific plausible causative KEs identified, this information is organized into linear cause–effect relationships, or pathways, as exemplified in Fig. 1: AOP7, from the AOPwiki, which describes a relationship between reduced aromatase levels with irregular ovarian cycling and impaired fertility (Nepelska et al. 2016). This, of course, is a simplified view of what is taking place in real life, yet serves its purpose well by highlighting KEs that can be measured by different tests, whether in silico, in vitro or in vivo. An AOP can also be branched should two or more KEs be essential for the progression to a downstream event, or if one KE leads to two or more downstream events. Likewise, individual KEs would necessarily be shared by many different AOs across biological systems and can thus serve as anchor points to create AOP networks. Examples of this can be seen in Figs. 2, 3, 4, 5 and 6.

It is important to note that AOPs are not specific to, or defined by, any specific chemical, but rather represents a biologically plausible chain of events that can be triggered by chemical exposure (Ankley and Edwards 2018). In this review, we will define a set of AOs for female reproductive health that we consider relevant to include in a test guideline, even though they may not yet be included as apical endpoints in any existing regulatory toxicity testing guideline. We will use these AOs as signposts for describing current knowledge about causal relationships between chemical exposures and female reproductive diseases.

To structure the AOPs into an accessible framework, we have opted to define key processes that are relevant for female reproductive disorders as starting points. We deliberately refer to all AOPs in this review as ‘putative’, as they are working concepts not yet included in the AOPwiki. This means that the numbering of pAOPs in this review are not to be confused with the numbering in the AOPwiki. From the processes considered crucial for ovarian and oocyte development such as meiosis, follicle assembly, folliculogenesis and atresia, we have defined putative AOs that could manifest from developmental disruption by chemical exposures. Our long-term goal is to build more complete toxicological safety assessments focused on female reproductive health and based on biological knowledge. These (p)AOPs can be used to confirm the utility of available test assays or prompt the development of new assays.

## Adverse outcome pathways as they relate to key processes in ovarian development and function

Ovarian development starts early in fetal life. It involves many essential processes, all of which can be disrupted and cause reproductive problems later in life. These essential processes include extra-gonadal specification of primordial germ cells, their migration to, and colonization of, the ovaries, and initiation of meiosis, alongside differentiation of organization of ovarian somatic cells and their niches. In some instances, chemicals can be triggers that disrupt these events and cause reproductive disorders that often manifest much later in life. Similarly, chemical exposures during adulthood can affect ovarian function or worsen the effects of developmental exposures. In the following, we propose ten pAOPs related to female reproductive disorders, originating from the disruption of fundamental processes in ovarian development or function.

### Adverse outcome pathways involving meiosis

Meiotic division is unique to germ cells and involves one round of DNA replication followed by two successive cell divisions—first the separation of homologous chromosomes (meiosis I), and then sister chromatids (meiosis II)—to yield haploid eggs or sperm. Meiosis initiates at 11–12 weeks of gestation in humans (Hunt and Hassold 2008), whereas it starts around 13 day post coitum (dpc) in mice (McLaren and Southee 1997). During fetal life, meiosis proceeds through the prophase I events synapsis and recombination, but is arrested in meiosis I until ovulation (Laisk et al. 2019). FSH and luteinizing hormone (LH) secreted by the pituitary from puberty onwards trigger exit from prophase I arrest, and primary oocytes complete the first meiotic division prior to ovulation, which will be discussed later. Here, we will focus on perturbed meiotic entry and progression in the fetal ovary as well as maintenance of meiotic arrest, as shown in the pAOP network, as presented in Fig. 2.

#### pAOP-1: “reduced retinoic acid levels lead to reduced follicle pool and impaired fertility in females”

This pAOP proposes that reduced retinoic acid levels will prevent oocytes from entering meiotic prophase I during early ovarian development. Ectopic activation of the retinoic acid-degrading enzyme CYP26B1 (*Cytochrome P450 family 26, subfamily B, member 1*) is proposed as one potential mechanism by which this occur, but the central step remains reduced retinoic acid regardless of upstream molecular regulation. Low or no retinoic acid in the ovaries will lead to a reduced number of primordial follicles and ultimately a diminished follicle pool. The rationale for including CYP26B1 as a MIE is that the enzyme is expressed in early stage ovaries prior to meiotic entry and CYP26B1 acts as the retinoic acid-degrading enzyme in the testes (Bowles et al. 2018). In other words, ectopic activation of CYP26B1 in the fetal ovaries would mimic the testis milieu and the female (XX) germ cells would initiate a developmental trajectory otherwise reserved for germ cells located in the testes, i.e., germ cell sex reversal (Spiller et al. 2017). This is the opposite to what would take place in the testes in the presence of a xenobiotic blocking CYP26B1 function, as previously depicted in a pAOP for disrupted testis development (Draskau et al. 2020).

##### Supporting evidence

Retinoic acid initiates meiosis by inducing expression of *Stra8* (*Stimulated by retinoic acid 8*), which subsequently triggers a chain of events required for meiotic progression; in mice (Bowles et al. 2006; Koubova et al. 2006) and in humans (Childs et al. 2011; Le Bouffant et al. 2010). Correct temporal retinoic acid-induced meiotic initiation thus seems crucial for the progression of oocyte development and ultimately a woman’s fertility (Bowles et al. 2006; Le Bouffant et al. 2010). Indeed, oocytes in vitamin A-deficient rats fail to enter meiosis by not upregulating *Stra8* (Li and Clagett-Dame 2009), showing an environmental vulnerability of the retinoid balance for germ cell development. In mice, the retinoic acid metabolizing enzyme CYP26B1 is an important factor in retinoic acid balance. *Cyp26b1* is expressed in both ovaries and testis until 12.5 dpc in mice, after which expression becomes testis specific. CYP26B1 is thought to be essential for degradation of retinoic acid and prevent meiotic entry of male germ cells (Bowles et al. 2006). Here, the proposal rests on the assumption that perturbed gonadal differentiation can cause ectopic expression of CYP26B1 in ovaries at stages when retinoic acid is needed to induce meiotic entry in female germ cells.

CYP26B1 appears to be expressed more robustly in human fetal ovaries and not downregulated during germ cell entry into meiosis, as in mouse ovaries (Childs et al. 2011; Frydman et al. 2017; Jørgensen et al. 2012), so the conserved role for this enzyme in germ cell development remains unclear. Germ cell differentiation in the human fetal ovary is asynchronous, with the presence of oogonia as late as second and third trimester (Frydman et al. 2017). This could indicate that CYP26B1 does have some meiotic inhibitory influence, or that, as suggested in above-mentioned studies, there are other factors, such as FGF9 (*Fibroblast growth factor 9*), involved in the sexually dimorphic initiation of meiosis versus mitotic arrest in male and female human gonads. Another explanation could be that the retinoic acid-synthesizing enzyme ALDH1A (*Aldehyde dehydrogenase 1 family, member A1*) is expressed in the ovary proper in humans, but in the adjacent mesonephros in mice. Thus, human ovaries have an intrinsic capacity to synthesize retinoic acid, which could trigger meiotic initiation at local sites within the ovary, as opposed to the anterior-to-posterior wave resulting from retinoic acid entering the gonads from the mesonephros at the anterior pole in mouse ovaries (Bowles et al. 2006; Childs et al. 2011).

##### Evidence for chemical triggers

In mice, in utero exposure to bisphenol A can result in reduced expression of *Stra8* at 17.5 dpc (Lawson et al. 2011). Exposure to di(2-ethylhexyl) phthalate can reduce expression of *Stra8* (gene and protein) at 13.5 dpc in mice (Zhang et al. 2015). The mechanism behind the effects remains unknown.

#### pAOP-2: “impaired GPER activity leads to reduced follicle pool and impaired fertility in females”

This pAOP proposes to link disrupted GPER (*G-protein-coupled Estrogen receptor 1*) signaling during initiation (fetal life) and maintenance (postnatal and adult life) of meiotic arrest and reduced follicle pool. The initiation, as well as maintenance, of meiotic arrest at prophase I requires high levels of cAMP, which is mediated through (GPCR (G-protein-coupled receptors) such as GPER. Thus, by regulating cAMP accumulation in oocytes, GPER signaling indirectly controls primordial follicle assembly and oocyte maturation (Pang and Thomas 2018). In the presence of estrogens, GPER signaling provides rapid non-genomic signaling. Thus, by disrupting estrogen-dependent GPER signaling, the follicle pool can be significantly reduced.

##### Supporting evidence

Activation of GPER is essential for estradiol-mediated primordial follicle formation (Pepling 2006; Wang et al. 2008) and GPER activity can affect oocyte maturation in mammalian and non-mammalian vertebrates (Li et al. 2013; Peyton and Thomas 2011). Reduced GPER activity during early stages of ovary development can reduce the size of the primordial follicle pool and thus affect the number of oocytes available at reproductive age. The maintenance of meiotic arrest in prophase I is mediated through GPCRs by activating adenylate cyclase to initiate the intracellular accumulation of cAMP and PKA (Protein kinase A) activation, ultimately inactivating the MPF (Maturation promoting factor) (Sen and Caiazza 2013). The ability of GPER to inhibit oocyte maturation is evidenced by the fact that oocyte maturation can be reversed with the GPER antagonist catecholestrogen (2- hydroxyestradiol-17beta) (Chourasia et al. 2015).

##### Evidence for chemical trigger

GPER-mediated responses are sensitive to both estradiol and EDCs with estrogenic potential. For example, bisphenol A has a higher binding affinity to GPER than to nuclear ERs (Estrogen receptors)) (Thomas and Dong 2006). It is evident that bisphenol A can increase GPER activity in male reproductive organs (Sheng and Zhu 2011; Vom Saal and Hughes 2005). Bisphenol A can also promote proliferation of ER-negative breast cancer cells, which suggests a proliferative effect of bisphenol A via the GPER pathway (Dong et al. 2011). Atrazine, a pesticide with reported estrogenic potential, can promote proliferation of ovarian cancer cells via GPER-EGFR induced Erk (Extracellular signal-regulated kinase) phosphorylation and expression of estrogen target genes, which may also stimulate ovarian cancer cell migration (Albanito et al. 2015). So too, the biocide DDT (1,1,1-trichlor-2,2-bis(4-chlorphenyl)ethan), or more precisely the p,p' and o,p' isomers of DDT and DDE, can both bind to GPER (Thomas and Dong 2006). In utero exposure to DDT has been associated with longer time to pregnancy in women, but solid evidence for DDT or related compounds affecting female reproductive function via the GPER pathway remains elusive.

#### pAOP-3: “disrupted meiotic division leads to impaired oocyte quality and impaired fertility in females”

This pAOP proposes to link disrupted synapsis and recombination via incorrect assembly of the synaptonemal complex with aneuploidy in oocytes and consequently reduced fertility in females. In humans, aneuploidy is found in ~ 20% of oocytes and the incidence increases with age. Approximately 35% of all miscarriages are aneuploid. Aneuploidy is the leading genetic cause of developmental and mental disabilities in pregnancies that survives to term (Hassold et al. 2007; Hassold and Hunt 2001).

##### Supporting evidence

Pairing of homologous chromosomes (synapsis) is mediated by the synaptonemal complex. One of the most important proteins for its establishment is SYCP3 (*Synaptonemal complex protein-3*). In *Sycp3* knockout mice, females are subfertile due to increased aneuploidy rates of oocytes (Yuan et al. 2002). The origin of aneuploidy in humans is complex, but the majority seems to occur already when the future mother of the child was herself a developing fetus. Typically, errors in the major events of prophase I—cohesion of sister chromatids, synapsis and recombination—can lead to incorrect chromosome segregation and pass this on to following generation (Hassold et al. 2007; Hassold and Hunt 2001). Hence, disrupted assembly of the synaptonemal complex and thereby incorrect synapsis causes aneuploidy or cell death (Bolcun-Filas and Schimenti 2012; Yuan et al. 2002; Zickler and Kleckner 2015).

##### Evidence for chemical triggers

In mice, exposure to bisphenol A during the final stages of oocyte growth can increase the rate of aneuploidy (Hunt et al. 2003), evidenced by a high frequency of synaptic defects, increased levels of recombination, as well as increased frequency of aneuploid oocytes and fetuses in adult females (Susiarjo et al. 2007). In Rhesus monkeys, significantly increased recombination is also seen in ooctyes after fetal exposure to bisphenol A, but only with a tendency towards synaptic defects (Hunt et al. 2012). Increased recombination has also been demonstrated in cultured human fetal oocytes exposed to bisphenol A; however, cytotoxicity was also observed at the concentrations, where effects were observed (Brieño-Enríquez et al. 2011). A recent study has also shown atrazine to disrupt meiosis and follicle formation in mice (Gely-Pernot et al. 2017).

### Adverse outcome pathways involving primordial follicle assembly

Primordial follicles form in the ovary by follicle assembly, a process that involves breakdown of germ cell nests, death of germ cells, and intrusion of pre-granulosa cells between the germ cells. In humans, follicle assembly is chaotic-looking and completed during fetal life, whereas in rats and mice it is initiated during fetal life but completed, in a more organized process than the human, around 6 days after birth (Grive and Freiman 2015; Pepling et al. 2010). Regardless of temporal differences in follicle assembly, however, the processes that are involved appear to be shared between species (Grive and Freiman 2015). Thus, rodents remain useful models for characterizing the molecular mechanisms underpinning both normal and abnormal primordial follicle assembly, as shown in Fig. 3.

#### AOP-4: “disrupted ESR and AHR signaling leads to disturbed primordial follicle formation and impaired fertility in females”

This pAOP network proposes that increased ERα and AHR (*Aryl hydrocarbon receptor*) signaling following EDC exposure results in accelerated primordial follicle formation and germ cell death, by dysregulating primordial follicle formation and intraovarian signaling. Ultimately, this leads to impaired adult fertility via compromised ovarian reserve. AHR is a ligand-activated transcription factor that is expressed in mouse and human ovaries and well-known for its role in xenobiotic metabolism. This pAOP is based on the weight of evidence for fetal ovarian development in the human, including disruption following exposure to maternal smoking in utero (Fowler et al. 2014) and underpinned by data from studies of model species considered more similar to the human than rat or mouse in terms of ovarian development, including baboon and hamster. The validity of the pAOP is supported by population-level evidence for the adverse effects of in utero exposure to maternal smoking on subsequent adult fertility of female human offspring (Ye et al. 2010).

##### Supporting evidence

The sequence from maternal smoking to fetal AHR activation, disturbed primordial follicle formation and reduced germ cell proliferation are well established (Anderson et al. 2015; Jurisicova et al. 2007; Matikainen et al. 2001; Tuttle et al. 2009). The increased AHR signaling in smoke-exposed fetuses is likely due to increased estrogen and estrogenic polycyclic aromatic hydrocarbon metabolites (Fowler et al. 2014), which leads to increased AHR signaling, (Tarnow et al. 2019). In addition, maternal smoking is associated with disturbed or reduced number of germ and gonadal somatic cells in the offspring (Lutterodt et al. 2009; Mamsen et al. 2010). The consequence of early estradiol and AHR activation would thus be early onset of primordial follicle formation (premature increase in germ cell VASA protein (gene *DDX4: DEAD-box helicase 4)* when germ cell OCT4 protein predominates in control (gene *POU5F1: POU class 5, homeobox 1*) and, given imbalanced proportions of germ and somatic cells, subsequently reduced primordial follicle pool establishment, leading to shorter reproductive lifespan and, ultimately, reduced fertility.

##### Evidence for chemical triggers

In smoke-exposed human fetuses and its ovaries, increased AHR and estrogen signaling has been associated with increased expression of steroid hormone receptor NR2F1 (*Nuclear Receptor subfamily 2, group F, member 1*), dysregulated CYP11A1 (*Cytochrome P450 family 11, subfamily A, member 1*) and FSHR (without alterations in circulating fetal FSH), increased NR2E1 (*Nuclear receptor subfamily 2, group E, member 1*) and HRK (*Activator of apoptosis harakiri*), reduced NOBOX (*Newborn ovary Homeobox-encoding gene*) and reduced inhibin/activin ratio (Fowler et al. 2014).

### Adverse outcome pathways involving folliculogenesis and follicular atresia

Folliculogenesis is the process by which activated primordial follicles grow and mature into competent oocytes in women of reproductive age, and involves several maturation stages (Gougeon 1986). Once activated, the surrounding granulosa cells proliferate to form a secondary follicle comprising two or more complete layers of granulosa cells. A liquid filled cavity, the antrum, will emerge within the granulosa cell layers to form an antral follicle that will continue to grow into a pre-ovulatory follicle. Once folliculogenesis has commenced it cannot be halted, nor reversed. The recruited follicle will either complete the maturation process or die by atresia. Folliculogenesis takes several menstrual, or estrus, cycles to complete. In humans it is estimated to take 6–9 months, whereas in mice, it takes at least 47 days (Gougeon 1986; Zheng et al. 2014).

Folliculogenesis comprises both a gonadotropin-independent and a gonadotropin-dependent growth phase. The gonadotropin-independent phase covers follicle activation and growth up to small antral stages. These stages can be seen even before activation of the hypothalamic–pituitary–gonadal axis during puberty (Abel et al. 2000). All follicles growing in the absence of gonadotropins, however, will eventually die by atresia. Conversely, small antral follicles will be stimulated to grow by FSH secreted from the pituitary gland after puberty. Once they become tertiary/Graafian follicles, they have reached a pre-ovulatory size, and a surge of LH will trigger final oocyte maturation, resumption of meiosis and subsequent ovulation.

Follicle growth is largely controlled by local autocrine and paracrine growth factors belonging to the TGFβ (*Transforming growth factor beta*) superfamily that are secreted by granulosa cells and oocytes of activated follicles. For example, activation of PI3K (*Phosphoinositide 3-kinase*) signaling leads to secretion of GDF9 (*Growth differentiation factor 9*) and BMP15 (*Bone morphogenetic protein 15*) from the oocytes, which in turn stimulate granulosa cell survival and proliferation through BMPR (Bone morphogenetic protein receptor)-mediated SMAD signaling. In addition, GDF9 is essential for mouse follicle development beyond primary stage (Dong et al. 1996). Granulosa cells secrete inhibins, activins and AMH (*anti-Müllerian hormone*), and AMH is involved in inhibition of the early stages of follicle growth (Carlsson et al. 2006; Durlinger et al. 2002; Visser et al. 2006). Notably, blood levels of AMH is proportional to the number of ovarian follicles, thus being a useful biomarker for assessing the ovarian reserve in women (Broekmans et al. 2008).

From the secondary follicle stage, the oocyte is surrounded by granulosa and theca cells. Theca cells produce androgens, which are transported to the granulosa cells and converted to estrogens by aromatase. Estrogens, in turn, stimulate a feedback loop that regulates androgen synthesis in the theca cells. This interplay between granulosa and theca cells is referred to as the “two-cell, two gonadotropin” theory (Ryan et al. 1968). It is also noteworthy that, in the ovary, androgens are not only precursors of estrogen production, but also important drivers of the transition from primordial to antral follicle (Franks and Hardy 2018; Lebbe and Woodruff 2013). In addition, estrogens play a key role in regulating the hypothalamic–pituitary–gonadal axis by exerting both negative and positive feedback on pituitary FSH and LH release. Thus, chemical influences on steroidogenesis will also affect folliculogenesis.

Follicular atresia is a process of follicular death that has features of both apoptosis and autophagy, and is believed to assure the ovulation of competent oocytes during reproductive age. However, certain environmental cues, such as chemical exposure, can disturb this tightly regulated balance and lead to clinical pathologies such as POI, which is characterized by, but not exclusively to, hormonal imbalances and a reduced ovarian reserve. In a recent review, the role for EDCs in inducing POI due to increased atresia in pre-antral follicles was highlighted (Vabre et al. 2017). One mechanism that may be involved in EDC-mediated follicle atresia is via AHR, as further explored below in pAOP 5 in Fig. 4.

#### pAOP-5: “disrupted AHR signaling leads to follicle atresia and premature ovarian insufficiency”

This pAOP proposes to link AHR signaling to premature ovarian insufficiency and impaired reproduction in women. The development of this AOP relies on evidence collected from mouse models and incorporates human data from a xeno-transplantation model (Matikainen et al. 2001) and women cohort data (Eskenazi et al. 2010). In the ovary, AHR also controls the expression of proapoptotic proteins BAX (*BCL2-associated X, apoptosis regulator*) and HRK in the pre-antral follicles leading to direct induction of apoptosis. The resulting loss of follicles leads to a decrease of the ovarian reserve and can result in premature ovarian insufficiency and early menopause.

##### Supporting evidence

The number of primordial ovarian follicles (i.e., the size of the ovarian reserve) determines fertility in females. Activation of AHR can induce death of immature and early growing follicles in mice leading to a reduction of the ovarian follicle pool (Jurisicova et al. 2007; Matikainen et al. 2001; Pru et al. 2009). This mechanism appears to be conserved between mouse and human (Jurisicova et al. 2007) and can lead to premature ovarian insufficiency and early menopause.

##### Evidence for chemical triggers

AHR-activating chemicals such as polycyclic aromatic hydrocarbons benzo[a]pyrene or dimethylbenz[a]anthracene, can induce primordial germ cell atresia and oocyte death via the intracellular apoptosis pathway in fetal and adult rodent ovaries, respectively (Matikainen et al. 2001, 2002; Neal et al. 2010). Polycyclic aromatic hydrocarbons found in tobacco smoke can activate AHR: offspring from mice exposed to polycyclic aromatic hydrocarbons (mimicking cigarette smoking) before pregnancy and during lactation displayed a significant reduction in primordial follicles (Jurisicova et al. 2007).

#### pAOP-6: “early PI3K activation leads to premature ovarian insufficiency”

This pAOP proposes to link activation of PI3K signaling in primordial follicles to premature ovarian insufficiency, leading to impaired fertility in adult females. The development of this AOP relies on evidence collected from mouse models and mechanistic information from both mouse and human models, showing that PI3K signaling controls early steps of follicle activation (Hsueh et al. 2015). This signaling pathway can be activated by various cell surface receptor tyrosine kinases, such as c-Kit, as well as by inhibition of PTEN (*Phosphatase and TENsin homolog deleted on chromosome 10*). PI3K signaling stimulates early follicle growth. This leads to more follicles exiting the pool of dormant follicles, which will reduce the ovarian reserve in the long term. Accelerated reduction of ovarian reserve can result in premature ovarian insufficiency and early menopause. Research has shown that this mechanism is conserved between mouse and human.

##### Supporting evidence

Experimental support is derived from a limited number of studies; however, it is well established that the number of ovarian follicles at birth determines the reproductive lifespan (Wallace and Kelsey 2010). Increased activation of the follicle pool will, therefore, shorten the reproductive lifespan. Activation of PI3K has been shown to induce activation of primordial follicles in mice (Reddy et al. 2008). Also, estradiol increases phosphorylation and decreases activity of PTEN in vitro (Scully et al. 2014).

##### Evidence for chemical triggers

Exposure to di-ethylhexyl phthalate and its metabolite mono-ethylhexyl phthalate has been shown to accelerate growth of isolated mouse ovarian follicles in vitro*,* as well as in mice in vivo with concurrent activation of PI3K in follicles measured as pAkt (Hannon et al. 2014, 2015). In mice, this results in premature ovarian insufficiency (Hannon et al. 2016).

#### pAOP-7: “decreased estradiol synthesis leads to alteration in ovarian cyclicity and impaired fertility in females”

This pAOP proposes to link inhibited steroidogenesis leading to lower estradiol levels and subsequently decreased ER action, which ultimately will result in modified ovarian cyclicity. Strong evidence comes from animal studies, which is supported by human epidemiological data and in vitro studies using human cells. Cyclic maturation of follicles is strongly dependent on coordinated hypothalamic–pituitary–gonadal axis function. Estradiol plays a key role in regulating the hypothalamic–pituitary–gonadal axis by exerting both negative and positive feedback on pituitary FSH and LH release (Moenter et al. 2020). Key events include inhibited estradiol production by ovarian granulosa cells and altered ERα activation in neuronal cells leading to alterations in ovarian cyclicity.

##### Supporting evidence

Correct ovarian cyclicity is essential for successful ovulation (Barbieri 2014). In addition, estrogen synthesized in the granulosa cells stimulates the maturation process of the primary follicle and endometrium for implantation. Disrupted ovarian cyclicity and modified follicle maturation can thus directly impact female fertility and is, therefore, considered an adverse health outcome. This is also observed in epidemiological studies. For example, women with shorter menstrual cycles were less likely to conceive and women with longer or shorter cycles were more likely to have spontaneous abortion (Small et al. 2006).

##### Evidence for chemical triggers

In adult rats, bisphenol A exposure can cause a decreased number of antral follicles and *corpora lutea*, as well as disrupted estrous cycle associated with delayed and lower amplitude LH surge (López-Rodríguez et al. 2019). Numerous studies link bisphenol A exposure to aromatase inhibition in granulosa cells and delay or suppression of LH surge resulting in modified ovarian cyclicity (Viguié et al. 2018). Di-(2-ethylhexyl) adipate can shorten the average length of the estrus cycle in rats (Wato et al. 2009), whereas acetyl tributyl citrate has been shown to do so in mice (Rasmussen et al. 2017). In humans, exposure to organophosphate pesticides has been related to a higher risk of irregular menstrual cycles and shorter menstrual bleed duration among Chinese women (Zhang et al. 2019). Consumption of phytoestrogen-rich soy foods increases menstrual cycle length in healthy premenopausal women (Hooper et al. 2009; Kumar et al. 2002). A reduction in LH concentrations was also found in healthy premenopausal women after a single dose of the phytoestrogen 8-prenylnaringenin (Rad et al. 2006).

#### pAOP-8: “decreased INSL3 synthesis leads to reduced follicle maturation and impaired fertility in females”

This pAOP proposes to link disrupted INSL3 (*Insulin-like factor 3*) signaling with follicle maturation. Evidence derives from both animal and human studies. In the adult ovary, INSL3 is mainly produced by the theca cells across species and acts by paracrine signaling on the oocytes. In rodents, loss of INSL3 compromises female fertility. In cow ovaries, INSL3 stimulates antral follicle growth in concert with LH and sex steroids. In humans, circulating INSL3 levels are elevated in women with PCOS.

##### Supporting evidence

INSL3 is expressed in ovarian theca cells (Bathgate et al. 1996; Ivell and Anand-Ivell 2018) and female *Insl3* knockout mice display reduced fertility (Nef and Parada 1999) or increased rate of follicular atresia and apoptosis (Spanel-Borowski et al. 2001). Studies suggest that somatic INSL3 act on germ cells via the G protein-coupled receptor RXFP2 (LGR8) and, by so doing, initiate oocyte maturation (Kawamura et al. 2004) by stimulating GDF9 production (Xue et al. 2014). In turn, this stimulates androgen synthesis in the theca cells (Xue et al. 2014). In cultured theca cells from cow ovaries, RNAi-mediated knockdown of INSL3 elicited a reduction in CYP17A1 (*Cytochrome P450 family 17, subfamily A, member 1*) mRNA and reduction in androstenedione production (Glister et al. 2013). In humans, circulating INSL3 levels may be a measure of theca cell function in women of reproductive age (Ivell and Anand-Ivell 2018), with elevated levels observed in women with PCOS (Anand-Ivell et al. 2013).

##### Evidence for chemical triggers

In adult rats, prenatal exposure before mating and during pregnancy to a mixture of brominated flame retardants downregulated *Insl3* and *Cyp17a1* expression in the ovary, but increased circulating androgen levels and caused increased number of (pre)antral follicles and antral follicle size in the dams (Lefèvre et al. 2016). There are numerous studies reporting disrupted INSL3 expression in testes following exposure to industrial chemicals such as phthalates leading to male reproductive tract anomalies (Anand-Ivell et al. 2018; Wang et al. 2019). It can be speculated that effects in the ovaries would be similar to effects seen in testes; however, direct evidence in the human female is lacking.

#### AOP-9: “disrupted AR activity leads to altered follicle growth and impaired fertility”

This pAOP proposes to link impaired activation of the AR (*Androgen receptor*) to adverse effects on reproductive capacity. The development of this AOP relies on evidence collected from rodent and pig models. The key events include impaired AR activation through AR antagonism followed by impaired follicle growth and ovulation.

##### Supporting evidence

AR is expressed in granulosa cells and is involved in the regulation of follicle activation and growth, since androgens can increase granulosa cell responsiveness to FSH (Franks and Hardy 2018; Lebbe and Woodruff 2013). Testosterone exposure in mice induces primordial follicle activation through both PI3K pathway-activation (Yang et al. 2010) and insulin-like growth factor 1 (IGF-1) signaling (Vendola et al. 1998, 1999). Androgen exposure induces growth of pre-antral and antral follicles, and mice lacking AR display impaired follicle maturation (Franks and Hardy 2018; Lebbe and Woodruff 2013). A study using cultured mouse follicles suggests that androgen homeostasis in the developing pre-antral and antral follicle is necessary to ensure optimal growth, steroidogenesis, and oocyte maturation (Lebbe et al. 2017).

##### Evidence for chemical triggers

Many EDCs are considered anti-androgenic based on their ability to block AR action in developing male rodents (Gray et al. 2006; Kortenkamp and Faust 2010; Schwartz et al. 2019; Vinggaard et al. 2008). Neonatal exposure to the AR antagonist flutamide delays initiation of folliculogenesis and reduces ovarian cell proliferation in pigs. In vitro, treatment of mouse pre-antral follicles with the AR antagonist enzalutamide (MDV) reduces follicle growth and counteracts the DHT-stimulated transition to antral follicles (Lebbe et al. 2017).

#### pAOP-10: “DNA methylation in LHCGR promoter leads to disturbed ovulation and impaired fertility in females”

This pAOP proposes to link DNA methylation in the transcription start site of the LHCGR (*Luteinizing hormone/choriogonadotropin receptor*) gene to impaired female fertility. LHCGR, also known as LH receptor, is necessary for triggering processes leading to ovulation and meiotic maturation of oocytes. The development of this AOP relies on evidence collected from zebrafish and mouse, and LHCGR appears to have evolutionary preserved roles.

##### Supporting evidence

Loss of signaling through the LHCGR cascades can cause atresia of mature oocytes in zebrafish (Migliaccio et al. 2018; Santangeli et al. 2016) and anovulation in mouse and human (Arroyo et al. 2020). For instance, increased DNA demethylation of LHCGR is observed in DHEA-induced PCOS mouse model (Zhu et al. 2010) and human granulosa cells of women with PCOS (Sagvekar et al. 2019).

##### Evidence for chemical triggers

Bisphenol A can disturb ovarian H3K4me3 and H3K27me3 patterns in the transcriptional start site of the LHCGR gene promoter, leading to downregulation of its transcript, as well as the expression of its downstream target PGRMC1 (*Progesterone receptor membrane component 1*). Increased level of atresia and complete block of ovulation was observed in adult zebrafish after 3 weeks of exposure to bisphenol A (Santangeli et al. 2016). PGRMC1, in turn, is involved in oocyte meiosis as its gene disruption in zebrafish leads to reduced response to progestin and decreased number of oocytes undergoing meiotic maturation (Wu et al. 2018).

## Test methods

A big challenge with developing AOPs for female reproductive disorders is the many knowledge gaps with respect to disease etiologies, not least at the mechanistic level. The AOP concept builds on the idea that we can improve both speed and accuracy of chemical testing by relying more on alternative test methods than on experimental animal testing. This requires, and will stimulate, the development of new alternative test methods, being it in silico, in chemico or in vitro, as well as improvement of endpoints in existing in vivo OECD-approved test guidelines for regulatory testing.

In vivo toxicity test guidelines include examination of ovarian weights and histology, estrous cyclicity and examination of pregnancy rate, fertility and pup survival, which can be considered quite crude and non-sensitive endpoints. The OECD guidance document 150 describes a *Conceptual Framework for Testing and Assessment of Endocrine Disrupters* that helps organize available test methods at different levels (L1-5) of biological organization to determine additional testing needs or conclude about the potential endocrine disrupter action of a chemical (OECD 2018a). Lower levels include non-test and in vitro assays, and higher levels include in vivo information with increasing degree of complexity. This document provides guidance for how to interpret data obtained from OECD Test Guidelines and other standardized test methods that can be used to evaluate chemicals for endocrine disruption primarily on those operating via estrogen, androgen, thyroid, steroidogenesis (EATS) modalities. In this review, we have included pAOPs that do not necessarily follow a classical endocrine mode of action. This would include those for disrupted meiosis (Fig. 2) and ectopic PI3K activation (Fig. 4). Nevertheless, they are equally important with regard to test strategies to prevent adverse reproductive outcomes, as the end result is the same: compromised female reproductive health and function. Also, our ten proposed pAOPs all comprise a set of KEs that do not necessarily correspond with a current OECD Test Guideline. In fact, a main goal of the FREIA project is to use AOPs to explain the mechanisms underlying chemically-induced female reproductive disorders and to highlight areas, where we should focus on improving or developing new test assays (https://www.freiaproject.eu). Below, we briefly present the current assay status for some of the most recurrent KEs in the pAOPs presented in this review.

### Key events related to meiosis

Testing for disrupted meiosis, or germ cell toxicity in general, is a challenge. This is particularly true for female germ cells, as they are much more cumbersome to harvest than their male counterpart, sperm. There are some interesting models being developed in non-mammalian systems, including yeast, fruit fly and flatworm (Ferreira and Allard 2015) that could potentially be adapted to study disrupted meiosis, depending on what modalities are being looked at. This could reduce reliance on in vivo KE, for instance measuring fertility rates in rodents. Since retinoic acid is a key initiator of germ cell meiosis, quantitative assays measuring retinoic acid levels in in vitro and in vivo samples are obvious choices; and several assays are already available (Grignard et al. 2020). As for CYP26B1, or CYP26A1, simply measuring ectopic gene or protein expression in fetal ovaries would suffice.

Regarding disrupted assembly of synaptonemal complex (pAOP 3), we are not aware of any current in vitro assays, or assays that could be adopted or modified to become fit-for-purpose. Effects on the synaptonemal complex and recombination are highly germ cell-specific and require cells to be analyzed while in meiosis I. SYCP3 and MLH1 (MutL homolog 1) immunolabelling can be performed to assess synapsis and recombination levels in in vivo studies, as done by others (Hunt et al. 2012; Susiarjo et al. 2007). While this is technically feasible, it is probably too laborious and might not be attainable to include in guideline protocols. However, analysing the aneuploidy rate in ovulated oocytes could provide the indirect measure to estimate the effect of EDCs on the fidelity of the meiotic segregation of chromosomes.

### Key events related to receptor interactions

Several of the pAOPs include KEs related to receptor interaction, for which there are already several assays and test methods available. For AR inhibition, the stably transfected AR-EcoScreen™ cell line can be used to test both agonism and antagonism of human AR by following OECD test guideline 458 (OECD 2016b). Similarly, there is an OECD performance based test guideline assay to study (ant)agonism at the ERα (OECD 2016a). We are currently developing an ERβ assay in the FREIA project using a high-content imaging platform. An additional assay of potential interest in relation to estrogenic activity is a fluorescent *vtg1* (*vitellogenin 1*) enhancer knock-in Zebrafish model recently developed (Abdelmoneim et al. 2020). For AHR (ant)agonism, several assays exists (Laier et al. 2003); however, at the time of writing they are not included in any OECD test guideline.

### Key events related to steroidogenesis

For measuring changes in steroid sex hormone synthesis, the already validated in vitro OECD test guideline H295R steroidogenesis assay (OECD TG 456) is an obvious choice (OECD 2011). It is in level 2 of the conceptual framework providing data about selected endocrine mechanisms and pathways (OECD 2018a). This cell line is derived from an adrenocortico carcinoma from a female patient and produces all of the steroid hormones in the classical steroidogenic pathway. The H295R steroidogenic assay enables the measurement of steroid sex hormone levels from the culture medium, as well as cellular expression level of steroidogenic enzymes. The gonads, however, display different pathways for steroidogenesis, including an alternative or “backdoor” pathway (Marti et al. 2017). This means that assays using theca or granulosa cells might be more relevant to detect disrupted ovarian steroidogenesis. We are currently investigating whether the current H295R steroidogenic assay is sufficient to capture potential effects of EDCs on the more complex, ovarian two-cell collaboration in steroidogenesis that is essential for ovarian estradiol production. It is conceivable that systems using intact ovaries or multiple cell types may reveal effects not detectable using an adrenal assay.

When addressing steroidogenesis, it might be useful to study interactions with specific steroidogenic enzymes, such as CYP17a1 or CYP19 (aromatase). The conceptual framework level 2 also includes an assay to study interactions with aromatase activity, i.e., aromatase assay (United States Environmental Protection Agency (US EPA) TG OPPTS 890.1200). While this test guideline has only been validated in the US, and not been accepted by the OECD, it is a commonly used assay in ED assessment in US. For interactions with CYP17A1, there are no current test guideline available. Previous studies have shown that this could be an added value to the conceptual framework, as CYP17 can be a target for EDCs and that this interaction differs from interactions with CYP19/aromatase (Roelofs et al. 2013).

### Key events related to folliculogenesis

Chemical disruption of folliculogenesis in vivo can be analyzed by quantification of the resting oocyte pool, growing ovarian follicles, as well as atretic follicles using histological sections. Such quantifications have been performed in several in vivo studies (Johansson et al. 2016; Mazaud et al. 2002; Patel et al. 2017), but it is a time-consuming process and would benefit from automation protocols. This is currently being explored in the FREIA project.

Specific assays need to be developed for KEs leading up to AOs that are measureable at the morphological level. Ex vivo models using exposed ovaries, or pieces of ovaries, at different stages of development can be useful for determining effect on each step of folliculogenesis, but no standard protocols are currently in focus for test method development (Hao et al. 2018). In culture systems using intact ovaries or multiple cell types, interactions with LHCR expression or INSL3 levels could be implemented. INSL3 levels can be measured in serum samples. It is worth investigating whether circulating INSL3 levels can be used as predictive biomarkers for female reproductive toxicity. Finally, activation of the PI3K-Akt signaling pathway is currently investigated by time-consuming quantification of the number of follicles positive for phosphorylated-Akt (pAkt) by immunostaining or by FOXO3 cytoplasmic translocation on histological slides from in vivo or ex vivo studies. However, cell-based assays specifically targeting this pathway could be beneficial in the long term.

## Perspectives

Although the AOP framework was proposed more than a decade ago, there are still not many ‘encyclopedic AOPs’ in the AOP-wiki. Notably, there is only one AOP (AOP7) that addresses female reproductive disorders in mammals (AOPwiki.org). Clearly, there is an urgent need to substantially update the AOP database and include more AOPs focusing on female reproductive toxicity. In this review, we have proposed ten pAOPs that we believe will be of use to researchers and regulators alike going forward. Neither of the pAOPs are yet included in the AOPwiki and much work remains before they become *encyclopedic*. From our synthesis of available literature, however, it is clear that alternative test assays are available to assess effects of chemicals on some early KEs. However, to use these alternative assays to predict AOs, plausible cause–effect relationships must be established to underpin any reliable data interpretation. Clearly, this requires an intensified effort towards describing female reproductive disease etiologies in the years to come if we are to reach our chemical safety assessment goals. The possibility of including more female KEs and AOs and thereby enhancing some of the in vivo Test Guidelines could be a way forward in the shorter term. So too, the discovery of robust biomarkers for female reproductive disorders, for instance the recently suggested CALB2 (Johansson et al. 2020), could prove very valuable additions to testing strategies as well as for clinical purposes.

Although the ten proposed pAOPs need additional work to be included in the AOPwiki, some of them rest on fairly solid ground. In particular, we envision pAOPs 1, 2, and 5 to gain rapid traction and, hopefully, populate the AOPwiki in the near future. This also extends to assay development or implementation of pre-existing assays by linking to shared KEs from other AOPs from separate biological systems. In fact, the premise of the current review is for more AOPs related to female reproductive disorders to be developed and included in the AOP-KB to serve as guides for risk assessors and chemical regulators under different jurisdictions.
